# Inhibition of JNK by compound C66 prevents pathological changes of the aorta in STZ-induced diabetes

**DOI:** 10.1111/jcmm.12267

**Published:** 2014-04-10

**Authors:** Yucheng Liu, Yonggang Wang, Xiao Miao, Shanshan Zhou, Yi Tan, Guang Liang, Yang Zheng, Quan Liu, Jian Sun, Lu Cai

**Affiliations:** a Kosair Children Hospital Research Institute at the Department of Pediatrics of the University of LouisvilleLouisville, KY, USA; b The First Hospital of Jilin UniversityChangchun, China; c The Second Hospital of Jilin UniversityChangchun, China; d The Chinese-American Research Institute, Wenzhou Medical UniversityWenzhou, China

**Keywords:** C66, diabetes, vascular damage, oxidative stress, JNK, Nrf2

## Abstract

Cardiovascular diseases as leading causes of the mortality world-wide are related to diabetes. The present study was to explore the protective effect of curcumin analogue C66 on diabetes-induced pathogenic changes of aortas. Diabetes was induced in male C57BL/6 mice with a single intraperitoneal injection of streptozotocin. Diabetic mice and age-matched non-diabetic mice were randomly treated with either vehicle (Control and Diabetes), C66 (C66 and Diabetes/C66) or c-Jun N-terminal kinase (JNK) inhibitor (sp600125, JNKi and Diabetes/JNKi). All three treatments were given by gavage at 5 mg/kg every other day for 3 months. Aortic inflammation, oxidative stress, fibrosis, cell apoptosis and proliferation, Nrf2 expression and transcription were assessed by immunohistochemical staining for the protein level and real-time PCR method for mRNA level. Diabetes increased aortic wall thickness and structural derangement as well as JNK phosphorylation, all of which were attenuated by C66 treatment as JNKi did. Inhibition of JNK phosphorylation by C66 and JNKi also significantly prevented diabetes-induced increases in inflammation, oxidative and nitrative stress, apoptosis, cell proliferation and fibrosis. Furthermore, inhibition of JNK phosphorylation by C66 and JNKi significantly increased aortic Nrf2 expression and transcription function (*e.g*. increased expression of Nrf2-downstream genes) in normal and diabetic conditions. These results suggest that diabetes-induced pathological changes in the aorta can be protected by C66 *via* inhibition of JNK function, accompanied by the up-regulation of Nrf2 expression and function.

## Introduction

Diabetic vascular complications are divided into microvascular and macrovascular disorders [1,2]. Macrovasculrar disorders include coronary artery diseases, atherosclerosis and peripheral vascular diseases. Unfortunately the development and progression of vascular complications in the diabetic patients remains unpreventable [1]. Therefore, more attention has been paid to the exploration of new ways to prevent and/or rescue diabetic complications.

Endothelial dysfunction is recognized as a major cause of diabetic vascular complications that may be mainly derived from diabetes-induced over-generation of oxidative stress, inflammation, fibrosis, apoptosis and proliferation. Oxidative stress means either over-generation of reactive oxygen and nitrogen species (ROS/RNS) or down-regulation of antioxidant defence system in the cell or tissue. Clinical trials with single or even two or three antioxidants have shown ineffective outcome for diabetic patients [3–6]; therefore, to up-regulate endogenous and multiple antioxidants may be a better approach for the prevention of diabetic vascular complications.

Transcription factor nuclear factor E2-related factor 2 (Nrf2) [7] acts as a master regulator of cellular detoxification responses [8]. Under physiological conditions, Nrf2 is generally localized in the cytoplasm, sequestered by its inhibitor Kelch-like ECH-associated protein 1 (Keap1) [9]. However, Keap1 as a molecular sensor undergoes chemical modifications in a series of reactive cysteine residues under oxidative or electrophilic stress conditions [10], allowing the release of Nrf2 from Keap1 and translocation of Nrf2 to nuclear [11]. Once in the nucleus, Nrf2 binds to antioxidant-responsive elements (ARE) in the promoter region of its downstream genes. Nrf2 downstream genes are mainly antioxidant and phase II enzymes such as NADPH quinineoxidoreductase (NQO1), haeme oxygenase-1, glutathione S-transferase, superoxide dismutase, catalase (CAT), and γ-glutamylcysteine synthase and other genes regulating the response to oxidative stress [12,13]. The Nrf2–ARE pathway is important in the cellular antioxidant defence system to protect the cell and tissue from oxidative stress 14; therefore, Nrf2 may be a potential prevention of and/or therapy for diabetic complications [15,16].

Curcumin is a naturally phenolic compound isolated as a yellow pigment from turmeric (Curcuma longa) and is extensively used in India, China and South East Asia [17]. Anti-inflammatory, antioxidant and antimicrobial effects of curcumin products have been widely investigated recently [18–20]. We have synthesized a few of novel curcumin analogues [21–24]. Among these analogues, C66 was found to effectively inhibit high glucose (HG)-induced inflammatory response and macrophage infiltration, resulting in a significant prevention of renal injury in diabetic rats [22–24]. However, whether C66 is able to prevent diabetes-induced vascular damage *via* up-regulation of Nrf2 and its downstream antioxidant proteins has not been addressed yet.

Therefore to define whether chronic treatment of diabetic mice with C66 can prevent the development and/or delay the progression of diabetes-induced aortic pathogenesis, we used a type 1 diabetic mouse model induced with single dose of streptozotocin (STZ). Diabetic and age-matched control mice were treated with C66 for 3 months. In addition, we previously found that C66's renal protection from diabetes was accompanied by a significant inhibition of c-Jun N-terminal kinase (JNK) 22; therefore, here some of diabetic mice and age-matched control mice were also treated with JNK inhibitor (JNKi, sp600125) for 3 months, to determine whether inhibition of JNK can result in a same protection as C66 against diabetes-induced aortic pathogenesis.

## Materials and methods

### Animals

C57BL/6J male mice, 8–10 weeks of age, purchased from the Jackson Laboratory (Bar Harbor, ME, USA), were housed in the University of Louisville Research Resources Center at 22°C with 12 hrs light/dark cycle as well as free access to standard rodent feed and tap water. Selection of male mice was to keep a consistent usage of the same gender as in our previous studies [23,24]. All experimental procedures for these animals were approved by the Institutional Animal Care and Use Committee of the University of Louisville, whose regulations are in compliance with the *Guide for the Care and Use of Laboratory Animals* published by the U.S. National Institutes of Health (NIH Publication No. 85–23, revised 1996). Type 1 diabetic mouse model was established by one intraperitoneal injection of STZ (Sigma-Aldich, St. Louis, MO, USA), dissolved in 0.1 M sodium citrate buffer (pH 4.5), at 150 mg/kg bodyweight, while age-matched control mice (Ctrl) only received the injection of same volume of 0.1 M sodium citrate buffer, to keep consistence with our previous studies [23,24]. Three days after STZ injection, mice with hyperglycaemia (blood glucose levels ≥250 mg/dl) were considered as diabetic (DM). Both diabetic and age-matched control mice were randomly treated by gavage with vehicle, C66 or JNKi for 3 months. Both C66 and JNKi (SP600125) were dissolved in 1% carboxyl methyl cellulose-Na as vehicle. All solutions were distributed at 5 mg/kg bodyweight every other day for 3 months.

### Aorta preparation and histopathological examination

After anaesthesia, mouse thoraxes were opened and the descending thoracic aortas were isolated carefully without rips or cuts. Aortic tissues were fixed in 10% buffered formalin overnight. The fixed tissues were cut into ringed segments (∽2–3 mm in length) ready to be dehydrated in graded alcohol series, clean with xylene, embedded in paraffin and sectioned at 5 μm thickness for pathological and immunohistochemical staining.

Paraffin sections from aortic tissues were dewaxed, incubated with 1× Target Retrieval Solution (Dako, Carpinteria, CA, USA) in a microwave oven for 15 min. at 98°C for antigen retrieval, followed by 3% hydrogen peroxide for 10 min. at room temperature and 5% animal serum for 60 min. respectively. These sections were then incubated with primary antibodies against plasminogen activator inhibitor-1 (PAI-1) at 1:100 dilution (BD Bioscience, San Jose, CA, USA), tumour necrosis factor-alpha (TNF-α) at 1:50 dilution (Abcam, Cambridge, MA, USA), 3-nitrotyrosine (3-NT) at 1:400 dilution (Millipore, Billerica, CA, USA), Nrf2 at 1:50 dilution, Phospho-SAPK/JNK (p-JNK) at 1:100 dilution (Cell Signaling, Boston, MA, USA) or Phospho-Nrf2 (p-Nrf2) at 1:200 dilution (Santa Cruz Biotechnology, Santa Cruz, CA, USA), overnight at 4°C. Afterwards, sections were washed with PBS, they were incubated with horseradish peroxidase conjugated secondary antibodies (1:100–400 dilutions with PBS) or Cy3-coupled goat antimouse IgG secondary antibody (1:100 dilution with PBS) for 1 hr in room temperature.

For colour development purposes, immunohistochemical staining sections were treated with peroxidase substrate 3,3′-diaminobenzidine kit (Vector Laboratories Inc., Burlingame, CA, USA) and counterstained the nuclei with haematoxylin while immunofluorescent staining sections were stained with 4′,6-diamidino-2-phenylindole (DAPI) at 1:1000 dilution to localize the nucleus.

### Sirius-red staining

Aortic fibrosis was detected by Sirius-red staining of collagen, as described in our previous study [5]. Briefly sections were stained with 0.1% Sirius-red F3BA and 0.25% Fast Green FCF. The stained sections were then assessed for the presence of collagen using a Nikon Eclipse E600 microscopy system (Tokyo, Japan).

### Terminal deoxynucleotidyl-transferase-mediated dUTP nick-end labelling (TUNEL) staining

TUNEL staining was performed with formalin-fixed and paraffin-embedded sections using Peroxidase In Situ Apoptosis Detection Kit S7100 (Millipore), according to the manufacture's instruction. Positively stained apoptotic cells were counted in at least five random microscopic fields of each section from the five animals in each experimental group with Nikon Eclipse E600 microscopy. Cells with TUNEL-positive nuclei were counted under high magnification 400× in six random fields from each mouse, and presented as TUNEL-positive nuclei per 100 vascular cell nuclei.

### Proliferating cell nuclear antigen (PCNA) staining

The PCNA staining kit (Invitrogen, Camarillo, CA, USA) was used for staining proliferating cells. Positively stained apoptotic cells were counted in at least five random microscopic fields of each section from the five animals in each experimental group with Nikon Eclipse E600 microscopy. Cells with PCNA-positive nuclei were counted under high magnification 400× in six random fields from each mouse, and presented as PCNA-positive nuclei per 100 vascular cell nuclei.

### Mast cell (MC) evaluation

Mast cells were examined with a modified toluidine blue stain, *e.g*. the acidified toluidine blue method described by Walsh et al. [25]. Sections that were treated by conventional dewax and rehydration were transferred to potassium permanganate solution for 2 min., following by tap-water rinse, to potassium disulphite solution for 1 min., following by tap-water rinse, and then to distilled water for 3 min. The sections were then placed in acidified toluidine blue solution for 5 min., followed by distilled water rinse, dehydrated rapidly and mounted. The quantities of MCs per view at magnification 400× relative of each group were presented. The quantities of MCs per view at magnification 400× in six random fields from each mouse were presented as MCs-positive cell numbers per view.

### Quantitative real-time PCR (qPCR)

Aortas were frozen with liquid nitrogen and stored at −80°C. Total RNA was extracted using the TRIzol Reagent (Invitrogen). RNA concentrations and purities were quantified using a Nanodrop ND-1000 spectrophotometer. First-strand complimentary DNA (cDNA) was synthesized from total RNA according to manufacturer's protocol (Promega, Madison, WI, USA). Reverse transcription was run in a Master cycler gradient (Eppendorf, Hamburg, Germany) at 42°C for 50 min. and 95°C for 5 min. with 0.5 μg of total RNA in a final volume of 20 μl that contained 4 μl 25 mM MgCl_2_, 4 μl AMV reverse transcriptase 5× buffer, 2 μl dNTP, 0.5 μl RNase inhibitor, 1 μl of AMV reverse transcriptase, 1 μl of dT primer and nuclease-free water. Primers of JNK, TNF-α, Nrf2, NQO-1 and CAT were purchased from Applied Biosystems (Carlsbad, CA, USA). The qPCR was carried out in a 20 μl solution including 10 μl of TaqMan universal PCR master mix, 1 μl of primer and 9 μl of cDNA with the ABI 7300 Real-Time PCR system. Data were expressed as fold increase compared with levels measured in controls by using the ΔΔ^Ct^ method and β-actin as a reference gene.

### Statistical analysis

Data were presented as mean ± SD (*n* = 5). Comparisons were performed by one-way anova for the different groups, followed by repetitive comparing Tukey's test with Origin 7.5 Lab data analysis and graphing software. Statistical significance was considered at *P* < 0.05.

## Results

### C66 prevented the pathological changes of aortas in diabetic mice

**Figure 1:**
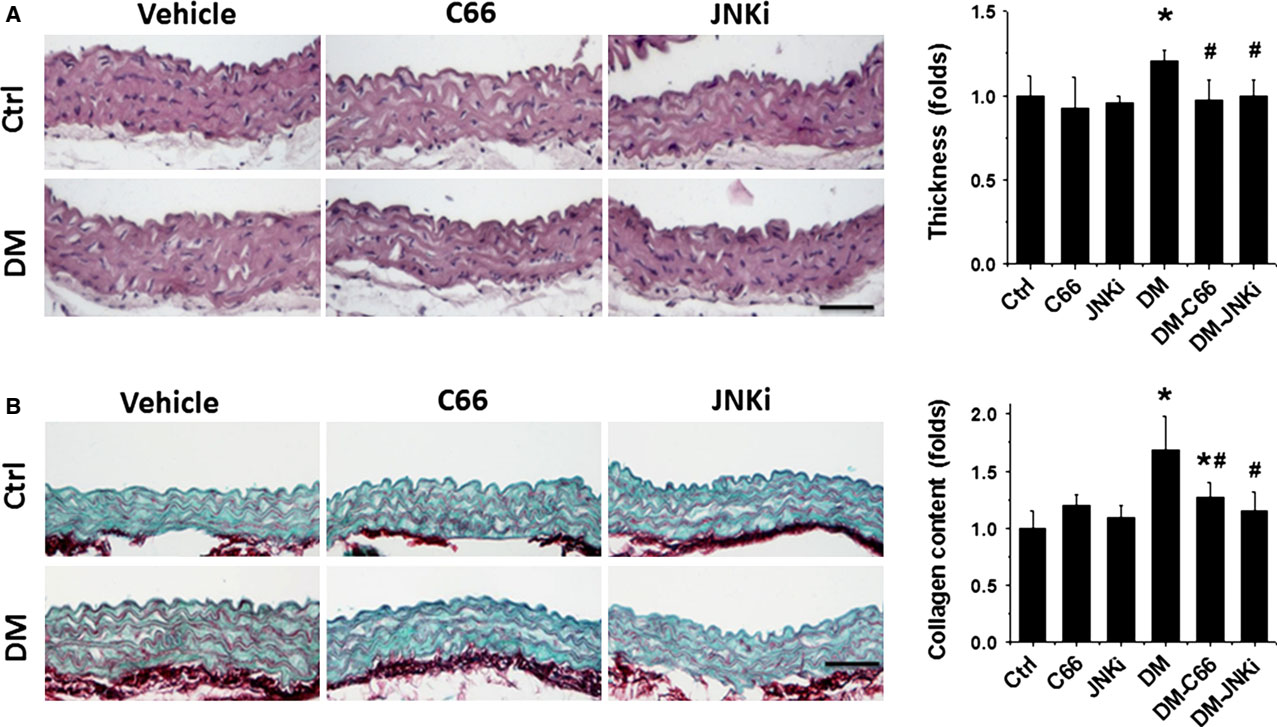
Protective effect of C66 on diabetes-induced aortic pathological changes. The pathogenic changes of aortas were examined by haematoxylin and eosin staining (A), and the dissimilarities in the accumulation of collagen was detected by Sirius-red staining (B), followed with semi-quantitative analysis. Data were presented as means ± SD (*n* = 5); *P < 0.05 *versus* Corresponding Ctrl; #*P* < 0.05 *versus* Corresponding DM; bar = 50 μM.

Microscopic examination of aortas with haematoxylin and eosin staining displayed that the tunica media thickness was significantly increased in DM mice (Fig.1A). Sirius-red staining revealed an increased collagen accumulation in tunica media of aortas in DM group (Fig.1B). These pathological changes seen in the aortas of DM mice were completely prevented by the 3-month treatment with C66 (Fig.1). An interesting result is that inhibition of JNK (JNKi) can capture a similar preventive effect to C66 (Fig.1).

### Diabetes significantly up-regulates JNK action, an effect that was prevented by C66

**Figure 2:**
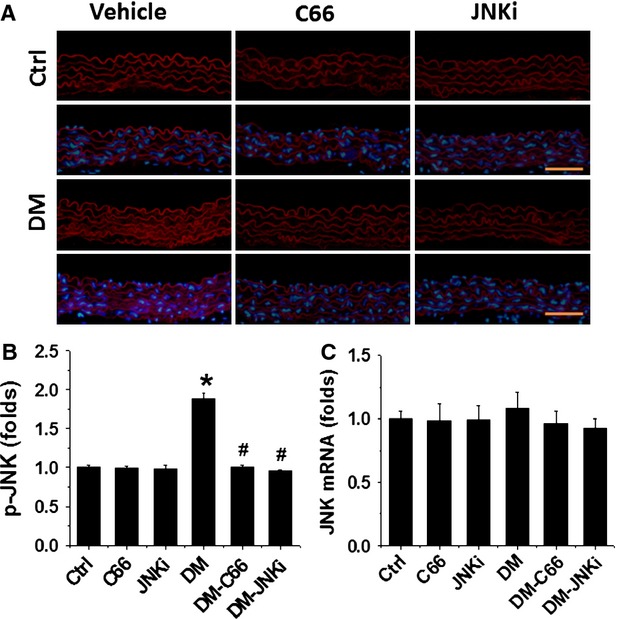
Effects of C66 on aortic JNK expression. The qualitative differences in the Phospho-JNK across all six groups by immunofluorescence staining (A), followed with semi-quantitative analysis (B) and real-time PCR for JNK expression at mRNA level (C). Data were presented as means ± SD (*n* = 5); *P < 0.05 *versus* Corresponding Ctrl; #*P* < 0.05 *versus* Corresponding DM; bar = 50 μM.

As activation of JNK has been suggested as possible mechanism for C66 to protect the kidney from diabetes [22] and Figure1 also showed a same preventive effect of JNK inhibition on diabetes-induced pathological changes of aortas as C66, next we examined whether diabetes activates JNK by immunofluorescent staining for JNK phosphorylation (Fig.2A and B) and RT-qPCR for JNK mRNA expression (Fig.2C). There was a significant increase in the expression of phosphorylated JNK in the aortic tunica media of DM mice, compared to control group. As expected, C66 indeed significantly inhibited the phosphorylation of JNK as did JNK inhibitor. However, there is no significant change in JNK mRNA expression among these groups.

### C66 protected from aortic apoptosis, proliferation, inflammation and oxidative damage in diabetic mice

**Figure 3:**
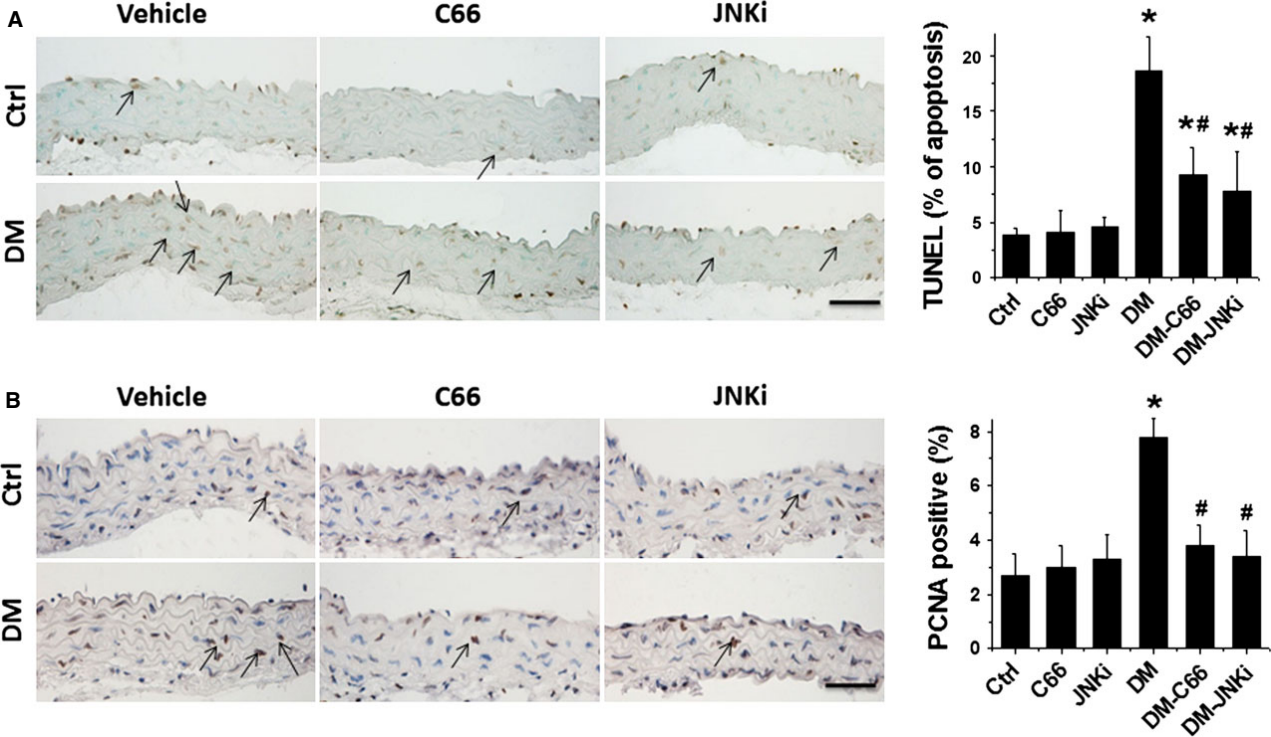
Protective effect of C66 on diabetes-induced aortic apoptosis and proliferation. The apoptotic cell was examined by TUNEL staining (A) and the proliferation of aortic tunica media was detected by proliferating cell nuclear antigen (PCNA) staining (B), followed with semi-quantitative analysis. Data were presented as means ± SD (*n* = 5); *P < 0.05 *versus* Corresponding Ctrl; #*P* < 0.05 *versus* Corresponding DM; bar = 50 μM.

We recently reported the induction of apoptotic cell death and cell proliferation in the aortas of DM mice [5], next study was to examine the effect of C66 and JNKi on the cell death and proliferation by TUNEL staining (Fig.3A) and PCNA immunohistochemical staining (Fig.3B), which showed significant increases in apoptotic cell death and proliferation in the aortas of DM mice, but not in the aortas of C66-DM or JNKi-DM mice.

**Figure 4:**
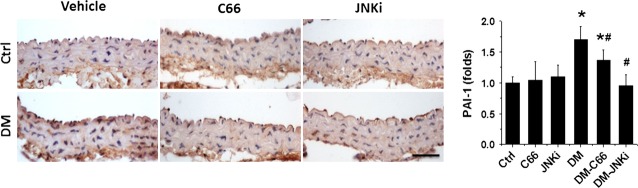
Protective effect of C66 on diabetes-induced aortic inflammation. The inflammation markers PAI-1 was examined by immunohistochemical staining, followed by semi-quantitative analysis. Data were presented as means ± SD (*n* = 5); *P < 0.05 *versus* Corresponding Ctrl; #*P* < 0.05 *versus* Corresponding DM; bar = 50 μM.

**Figure 5:**
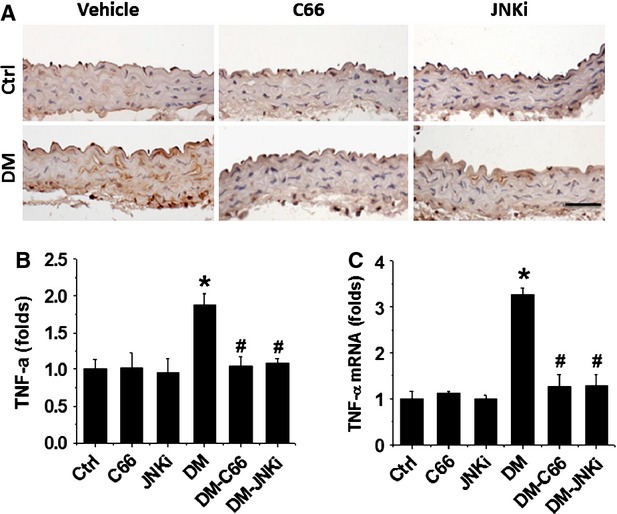
Protective effect of C66 on diabetes-induced aortic tumour necrosis factor-alpha (TNF-α) expression. Aortic expression of TNF-α was examined by immunohistochemical staining for its protein (A) expression in aortic tunica media, followed by semi-quantitative analysis (B) and real-time PCR for its mRNA level (C). Data were presented as means ± SD (*n* = 5). *P < 0.05 *versus* Corresponding Control; #*P* < 0.05 *versus* Corresponding DM; bar = 50 μM.

Given that both inflammation and oxidative damage are primary risk factors for the vascular endothelium remodelling, the expression of PAI-1 (Fig.4) and TNF-α (Fig.5 A and B) was examined with immunohistochemical staining, and the expression of TNF-α mRNA (Fig.5C) was examined with RT-qPCR, which showed a significant increase in PAI-1 and TNF-α (both protein and mRNA level) in the aortas of DM mice, but not C66-DM or JNKi-DM mice.

**Figure 6:**
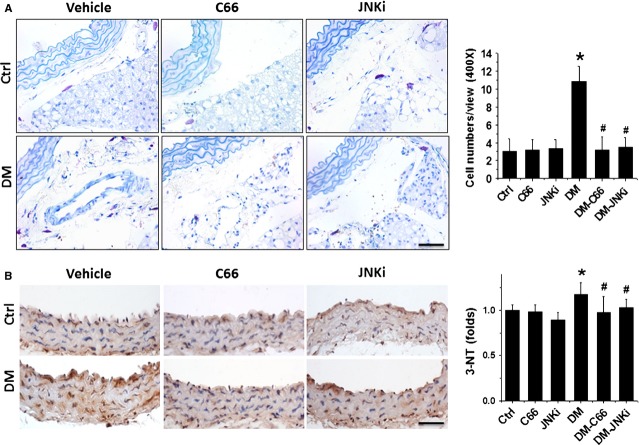
Effects of C66 on diabetes-induced mast cells evaluated around the aortic and oxidative stress damage. The mast cells around the aortic were identified by toluidine blue staining (A). Magnification at 400 ×  followed semi-quantitative analysis. The oxidative stress damage was examined by immunohistochemical staining for the accumulation of 3-NT (B), followed with semi-quantitative analysis. Data were presented as means ± SD (*n* = 5); *P < 0.05 *versus* Corresponding Ctrl; #*P* < 0.05 *versus* Corresponding DM; bar = 50 μM.

A body of evidence has indicated that MCs play a critical role in initiating local inflammation by secreting inflammatory cytokines [26,27]. By biochemical staining, we demonstrated a significant increase in MCs in the connective tissues around aorta, predominantly located round microvessels (Fig.6A). Treatment with either C66 or JNKi completely prevented the increase in MCs in the connective tissues around aorta.

It is known that inflammation and oxidative stress are reciprocal causes and outcome [28]. Immunohistochemical staining showed a significant increase in oxidative and nitrative damage, reflected by the increased accumulation of 3-NT, in the aortic tunica media of DM mice, but not C66-DM or JNKi-DM mice (Fig.6B).

### C66 Up-regulates aortic Nrf2 expression and transcription function

**Figure 7:**
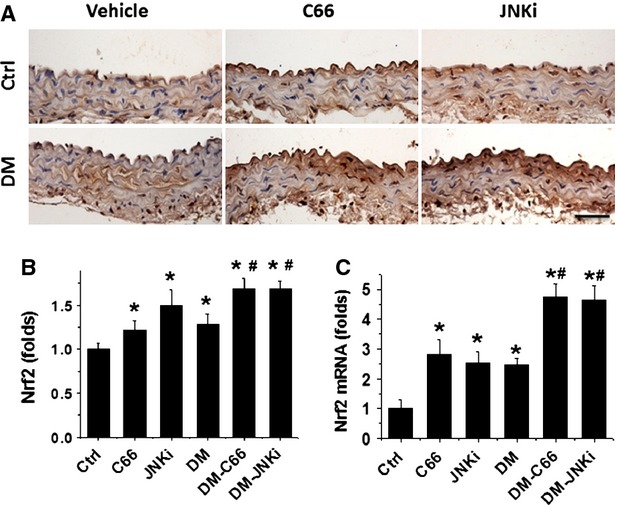
Effects of C66 on aortic Nrf2 expression. The expression of Nrf2 was examined by immunohistochemical staining for Nrf2 (A) expression at protein level in aortic tunica media, followed semi-quantitative analysis (B) and real-time PCR for its expression at mRNA (C) level. Data were presented as means ± SD (*n* = 5); *P < 0.05 *versus* Corresponding Ctrl; #*P* < 0.05 *versus* Corresponding DM; bar = 50 μM.

**Figure 8:**
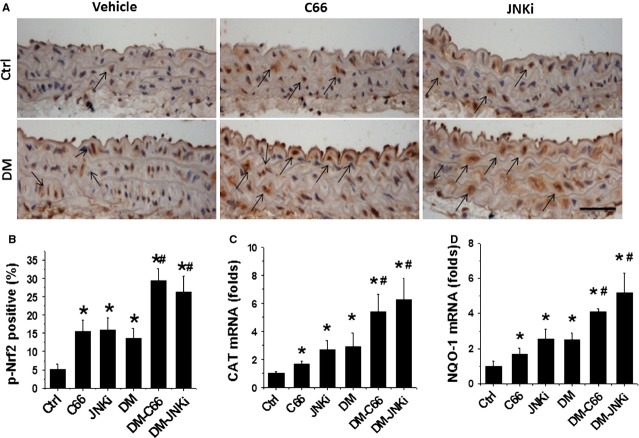
Effects of C66 on aortic Nrf2 function. Aortic expression of p-Nrf2 was examined by immunohistochemical staining (A), followed semi-quantitative analysis (B), and Nrf2-downstream genes CAT (C) and NQO-1 (D) expression was examined by real-time PCR at mRNA level. Data were presented as means ± SD (*n* = 5); *P < 0.05 *versus* Corresponding Ctrl; #*P* < 0.05 *versus* Corresponding DM; bar = 50 μM.

As oxidative stress was considered as the pivotal mediator for various cardiovascular complications of diabetic patients, we assume that the above pathological changes in the aortas of DM mice may all be attributed to the increased oxidative stress, and the protective effect by C66 or even JNKi on diabetes-induced aortic pathogenesis may be mediated by up-regulation of endogenous antioxidants; therefore the next study was to examine the expression and transcription of Nrf2. Figure7 showed that diabetes significantly increased the expression of Nrf2 protein (Fig.7A and B) and mRNA (Fig.7C) in the aorta of DM group compared to control. Diabetes also slightly up-regulated the expression of p-Nrf2 protein (Fig.8A and B) and the expression of Nrf2-downstream antioxidant genes: CAT (Fig.8C) and NQO-1 (Fig.8D). Both treatments with C66 and JNKi significantly and even synergistically increased Nrf2 expression (Fig.7) and function (Fig.8) in non-diabetic mice and diabetic mice respectively.

## Discussion

We have provided the first evidence to show the protective effect on diabetes-induced pathogenic changes in the aortic with C66 as a curcumin analogue. In STZ-induced diabetic mice, we found the significant increase in JNK phosphorylation, an effect that can be prevented by treatment with C66 as did JNK inhibitor. We also demonstrated that diabetes significant increased aortic wall thickness, fibrosis, inflammation, oxidative damage, apoptosis and proliferation, all of which were significantly prevented by treatment with C66 or JNKi. These results suggest that C66 prevents diabetes-induced pathogenic changes in the aortic *via* inhibition of JNK function.

Chronic inflammation plays an important role in the development and progression of various chronic pathogeneses, including oxidative stress, apoptotic cell death and cell proliferation, under diabetic conditions [29–32]. Curcumin has been shown for its important protection against various vascular injuries induced by HG, ageing, atherosclerosis and high blood pressure [33–37]. However, a few of these studies were done *in vitro* for looking for the protective effect of native curcumin on HG-induced changes [33,37]. Among the *in vivo* studies, the protective effect of native curcumin was observed only in those used with a very high dose of native curcumin, for instance either 2% food intake [36] or 100–200 mg/kg [35,38], but not in those used with a relative low dose of curcumin such as 50 mg/kg bodyweight [39]. In the present study, however, we demonstrated the increased expression of PAI-1 (Fig.4) and TNF-α (Fig.5) as inflammation markers in the aorta of DM group, along with significant increases in aortic oxidative damage, apoptotic cell death, cell proliferation and remodelling. All these pathological changes were significantly reversed in diabetic mice treated with 5 mg/kg bodyweight C66, a curcumin analogue [22,40]. Taken together of our findings with previous studies [33–38], we can conclude that curcumin is able to protect the cardiovascular system from oxidative damage in various oxidative stress models.

Mast cells are recognized as active participants in mediating a wide range of reactions including autoimmunity, inflammation and infection [41]. Mast cells are tissue-specific and respond to different stimulants in different tissues [41]. The cells function by producing secretory granules that release an assortment of bioactive molecules including cytokines, chemokines and proteases, such as chymase and tryptase, into the surrounding tissues, leading to tissue remodelling [41]. Mast cells are sensitive to many environmental stressors and those specific to diabetes [41–43]. Therefore, recent studies showed that mast cells play an important role in cardiovascular remodelling under diabetic conditions [43,44]. We showed here for the first time that C66 can significantly reduce diabetes-increased the filtration of mast cells in the connective tissue surrounding aorta along with a prevention of aortic pathological changes. This association may explain the important role of mast cells in the development of aortic inflammation, cell death and remodelling.

Curcumin has been reported to activate Nrf2 expression and function in a variety of conditions [45–47]. Thus the mechanism by which C66 prevents diabetes-induced aortic pathogenesis may be associated with the up-regulation of Nrf2. In previous studies from our own and others, Nrf2 was found to play important role in preventing diabetes-induced aortic damage [5,48,49] and cardiac or renal damage [15,50–52]. Here, we demonstrated that C66 treatment also significantly increased Nrf2 expression and function (Figs7 and 8). Increased Nrf2 function was reflected by the increased activation of Nrf2: Nrf2 Phosphorylation and its downstream antioxidant genes: CAT and NQO-1 (Fig.8). Ungvari and colleagues used Nrf2 gene knockout (Nrf2-KO) mice to show that increases in endothelia ROS levels and endothelial dysfunction induced by feeding with high-fat diet were significantly greater in Nrf2-KO mice compared with wild-type mice [53]. It should be noted that in the present study, diabetes also increased aortic Nrf2 expression (Fig.7) and its downstream CAT and NQO-1 gene expression (Fig.8), which suggests that Nrf2 acts as a protective mechanism at certain early stages to be up-regulated with the attempt to protecting the tissue. At the present study, a mild increase in Nrf2 expression and even function may remain not enough to compensate the severe damage induced by diabetes; however, when C66 and JNKi treatment synergistically increased Nrf2 expression and function to effectively prevent diabetes-induced severe damage.

The novel finding of the present study is the JNK that mediates C66's aortic protection from diabetes. JNK is a key transcriptional regulator of inflammatory cytokines and also plays a critical role in regulating the NF-κB activity in various types of cell lines [54]. Specifically, JNK has been found to play a significant role in the expansion of abdominal aortic aneurysms and cerebral artery aneurysms [35,55,56]. The JNK signalling pathway is also involved in the apoptosis of vascular walls [56]. The inhibition of JNK not only prevents the development of an abdominal aortic aneurysm but also causes the regression of an established abdominal aortic aneurysm.

**Figure 9:**
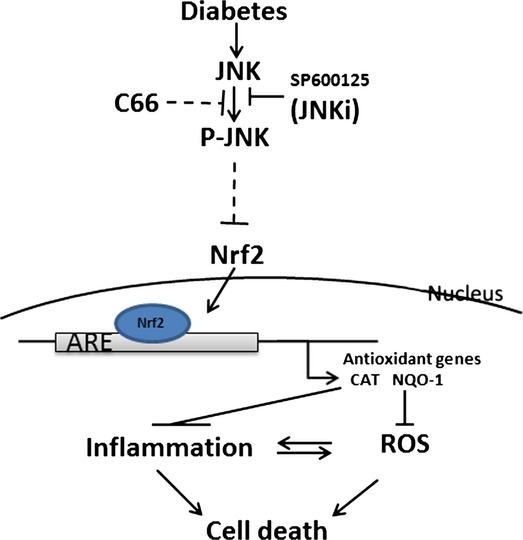
Schematic illustration for the protection of C66 against high glucose-induced inflammation and oxidative damage and consequently aortic remodelling *via* JNK inactivation.

There were also studies that have demonstrated the preventive effect of curcumin on cardiovascular diseases *via* inhibition of JNK [35,57–59]. To consistent with these previous studies, we also demonstrate that C66 is able to reduce the HG-induced inflammatory response, which was accompanied by inhibitory effects on JNK/NF-kB activation [22]. However, the present study has directly defined the role of JNK inhibition in preventing diabetes-induced aorta's pathogenesis, as illustrated in Figure9.

Interestingly we provided the evidence that inhibition of JNK also stimulates aortic Nrf2 expression and function in both control and diabetic mice, suggesting the inhibitory effect of JNK on Nrf2 expression and function under normal physiological and pathophysiological conditions. Although we do not know how JNK inhibits Nrf2 expression and function, our finding is in the line with a previous finding that high JNK activity with low Nrf2 expression and function; while inhibition of JNK resulted in up-regulation of Nrf2 expression and function [60]. However, the exact mechanism by which JNK inhibits Nrf2 expression and function in the physiological condition still needs to be further dissected in the future.

## Conclusions

In summary, we have investigated whether C66 can protect the aorta from diabetes using a type 1 diabetes mouse model for the first time. Treatment of diabetic mice with C66 for 3 months can almost completely reverse and/or prevent the progression of diabetes-induced aortic oxidative damage, inflammation, apoptosis and proliferation. Mechanism responsible for the protective effect of C66 is mediated by inhibition of JNK that may be also related to up-regulation of Nrf2 expression and function, as illustrated in Figure9. Although the detailed mechanism requires additional exploration, at least, the present study provides an interesting piece of evidence for the potential application of C66 to clinics for diabetic patients to prevent their cardiovascular complications *via* inhibition of JNK function.
